# Screening of Bioactive Metabolites and Biological Activities of Calli, Shoots, and Seedlings of *Mertensia maritima* (L.) Gray

**DOI:** 10.3390/plants9111551

**Published:** 2020-11-12

**Authors:** Kihwan Song, Iyyakkannu Sivanesan, Gunes Ak, Gokhan Zengin, Zoltán Cziáky, József Jekő, Kannan RR Rengasamy, O New Lee, Doo Hwan Kim

**Affiliations:** 1 Department of Bioresources Engineering, Sejong University, 209 Neungdong-ro, Gwangjin-gu, Seoul 05006, Korea; khsong@sejong.ac.kr; 2 Department of Bioresources and Food Science, Institute of Natural Science and Agriculture, Konkuk University, Seoul 05029, Korea; kimdh@konkuk.ac.kr; 3 Department of Biology, Faculty of Science, Selcuk University, 42130 Konya, Turkey; akguneselcuk@gmail.com (G.A.); gokhanzengin@selcuk.edu.tr (G.Z.); 4 Agricultural and Molecular Research and Service Institute, University of Nyíregyháza, 4400 Nyíregyháza, Hungary; cziaky.zoltan@nye.hu (Z.C.); jjozsi@gmail.com (J.J.); 5 Institute of Research and Development, Duy Tan University, Da Nang 550000, Vietnam; rengasamyrrajakannan@duytan.edu.vn; 6 Faculty of Environment and Chemical Engineering, Duy Tan University, Da Nang 550000, Vietnam; 7 Indigenous Knowledge Systems Centre, Faculty of Natural and Agricultural Sciences, North-West University, Private Bag X2046, Mmabatho 2745, North West Province, South Africa; 8 Department of Bioindustry and Bioresource Engineering, Sejong University, 209 Neungdong-ro, Gwangjin-gu, Seoul 05006, Korea; onewlee@sejong.ac.kr

**Keywords:** axillary shoot multiplication, antioxidant activity, conservation, plant growth regulators, enzyme inhibition, flavonoids, phenolics

## Abstract

*Mertensia maritima* (L.) Gray is threatened with extinction owing to climate change, poor seed germination, and ocean warming. In vitro explant-culture is used for ex situ preservation and plantlet massive production. In vitro cell and organ cultures serve as an alternative plant material source to investigate the biological activities and phytochemical profiles of rare plants. We aimed to develop an efficient callus and shoot production protocol and investigate bioactive metabolites, antioxidants, and enzyme inhibitory potential of *M. maritima* calli, shoots, and in vivo seedlings. The effects of combinations of different plant growth regulators, 6-BA (N^6^-benzyladenine), 6-KN (Kinetin), TDZ (Thidiazuron), and NAA (1-Naphthylacetic acid), in MS (Murashige and Skoog) nutrient medium were studied. The highest callus proliferation was obtained after 5-week cultivation over a 16-h photoperiod on growth medium MS enriched with 4 µM each of 6-BA and NAA. The medium with 2 µM 6-BA and 4 µM 6-KN had the best shoot induction rate (91.1%) with a mean of 13.4 shoots. The combination of two cytokinins (6-BA and 6-KN) was found to be effective in *M. maritima* shoot regeneration. The rooting frequency was 100% in ½ MS with Indole-3-butyric acid (IBA 2 µM). The number of detected compounds and chemical composition in the *M. maritima* shoots and seedlings extracts were similar. The total amount of phenolics in the shoots was 216.4% and 369.5% higher than in seedlings and calli, respectively. The total amount of flavonoids in the shoots was 241.1% and 429.3% higher than in seedlings and calli, respectively. The best antioxidant activity was obtained in the shoots, followed by seedlings and calli. However, the order was seedlings > calli > shoots regarding metal chelating ability. The strongest acetylcholinesterase inhibition properties were obtained in the calli, followed by seedlings and shoots. However, the tested samples can be ranked as seedlings > shoots > calli in butylcholinestrase inhibition assay. This study is the first report on the enzyme inhibitory effects of *M. maritima* extracts, providing valuable contributions to the scientific community.

## 1. Introduction

The genus *Mertensia* of the family Boraginaceae comprises 62 species of perennial herbs widely distributed in Europe, North and Central America, and Northern Asia [1]. Several *Mertensia* species are traditionally used to treat tuberculosis, venereal diseases, and whooping cough [2]. Pyrrolizidine alkaloids such as lycopsamine and intermedine were found in *M. bakeri* (Greene) and *M. ciliata* (James) G. Don [3]. *M. maritima* (L.) Gray, also recognized as an oyster plant, is largely found in the northern hemisphere. It has striking blue-green leaves and blooms from June to September with pink and blue flowers; thus, it has high ornamental value. The fresh leaves, taproots, and flowers of *M. maritima* are eaten by the Iñupiat of Alaska [2]. However, the presence of hepatotoxic pyrrolizidine alkaloids in the tissues of *M. maritima* has not yet been disclosed. It contains several bioactive metabolites such as carotenoids, phenolic acid, terpenoids, tocopherol, fatty acids, and volatile compounds [4,5,6], which are well-known to have many biological activities. In addition, the pharmacological activities of *Mertensia* species have not yet been documented.

**Figure 1:**
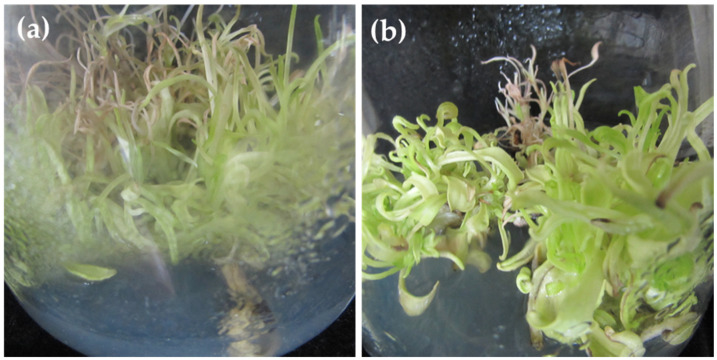
(**a**) Shoot tip necrosis occurred after 5-week cultivation over a 16-h photoperiod in a culture medium containing MS (Murashige and Skoog) + 1 µM NAA (1-Naphthylacetic acid) + 4 µM TDZ (Thidiazuron) [6]. (**b**) Hyperhydricity occurred after 5-week cultivation over a 16-h photoperiod in a culture medium containing MS + 1 µM NAA + 2 µM TDZ [6].

*M. maritima* is at risk of extinction owing to climate change and ocean warming [7]. Although the cultivation of oyster plants in nurseries is often difficult [7], it has been successfully grown in southwestern France and Northern Scotland [4]. *M. maritima* is naturally propagated by seeds. However, the mass production of *M. maritima* using conventional methods is hampered because of its poor seed germination. Therefore, an alternative mass production method for *M. maritima* would be valuable. In vitro explant-culture methods are effectively used for ex situ germplasm maintenance and the massive production of plantlets and bioactive metabolites. Recently, the in vitro micropropagation of *M. maritima* was reported [6]; the authors used TDZ (Thidiazuron) for micropropagation. However, the mass production of healthy *M. maritima* shoots is affected by TDZ. Continuous TDZ exposure resulted in shoot tip necrosis and hyperhydricity (Figure 1a,b). Hence, the identification of an efficient plant growth regulator (PGR) is necessary for the in vitro multiplication of *M. maritima* shoots.

Several bioactive metabolites such as allantoin, rabdosiin, rosmarinic acid, all-*E*-lutein, all-*E*-β-carotene, all-*E*-violaxanthin, 9-*Z*-neoxanthin, (*Z*)-β-carotene, all-*E*-zeaxanthin, α-tocopherol, α-linolenic acid, palmitic acid, linoleic acid, γ-linolenic acid, stearidonic acid, stearic acid, lignoceric acid, behenic acid, and arachidic acid were obtained from *M. maritima* callus and shoot cultures [5,6]. However, the screening of bioactive metabolites, including phenolics, in *M. maritima* calli, shoots, and in vivo seedlings, has not yet been reported. Thus, the aims of this work were to (a) determine the effects of PGRs on callus and shoot proliferation, (b) assess the bioactive metabolite profile of in vitro-developed calli and shoots and in vivo seedlings, and (c) estimate the antioxidant capacity and enzyme inhibitory activity of *M. maritima*.

## 2. Results

### 2.1. In Vitro Propagation

#### 2.1.1. Callus Induction

**Figure 2:**
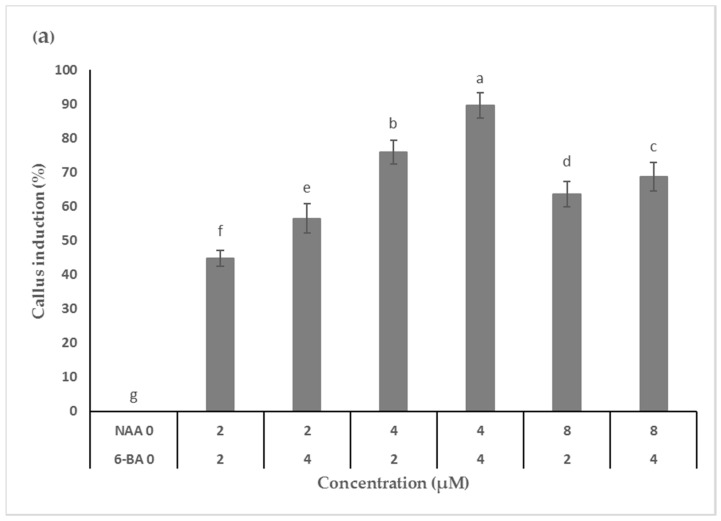

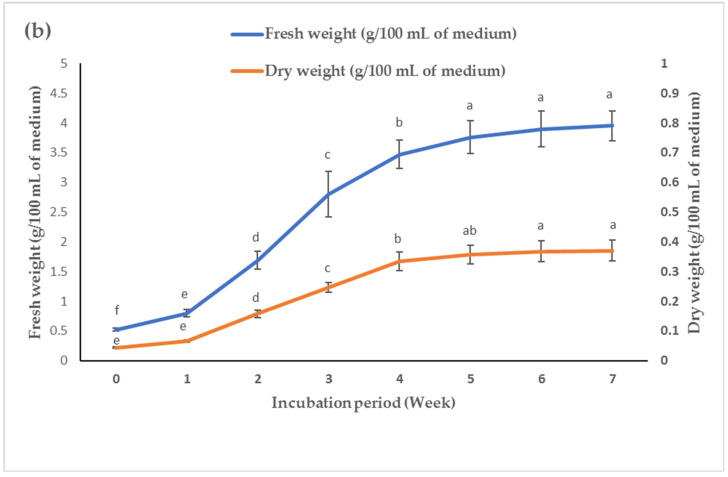
Influence of PGRs on callus induction and growth. (**a**) Effect of 6-BA (N6-benzyladenine) and NAA (1-Naphthylacetic acid) combination on callus induction; (**b**) growth of callus culture in MS (Murashige and Skoog) medium with 4 µM each of 6-BA and NAA. Different letters in the graph indicate significant differences at *p* < 0.05 (DMRT).

**Figure 3:**
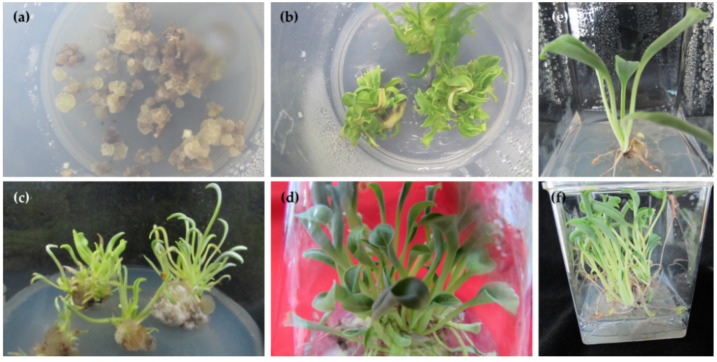
Micropropagation of *M. maritima.* (**a**) Callus formed on a culture medium MS (Murashige and Skoog) + 4 µM of each 6-BA (N^6^-benzyladenine) and NAA (1-Naphthylacetic acid) after 5 weeks; (**b**) multiple shoots regenerated on a culture medium MS + 2 µM 6-BA and 4 µM 6-KN (Kinetin) after 4 weeks; (**c**) multiple shoots regenerated on culture medium MS + 8 µM 6-KN and 1 µM NAA after 4 weeks; (**d**) shoots elongated on PGR-free MS medium after 5 weeks; tooted shoots on half-strength medium with 2 µM Indole-3-butyric acid (**e**) after 3 weeks and (**f**) 6 weeks of cultivation.

Leaf explants of *M. maritima* inoculated on control medium (MS medium devoid of hormones) failed to develop calli after 5 weeks of cultivation. Calli were induced in the inoculated explants on medium with combinations of 6-BA (N^6^-benzyladenine) and NAA (2-(1-naphthyl) acetic acid) (Figure 2a). Callus initiation was detected within 2 weeks of cultivation. Of the six different combinations of 6-BA and NAA studied, 4 µM of each 6-BA and NAA was found to be the most useful for callusing with 89.6% frequency (Figure 3a). Calli induced on this media thrived compared with those induced using other treatments; therefore, the above medium was selected to study callus growth. The lowest percentage of callus initiation (44.7%) was noticed in the medium with 2 µM of 6-BA and NAA (Figure 2a). Light green, friable calli obtained from leaf explants of *M. maritima* were inoculated on medium containing 6-BA and NAA each at 4 µM, and growth was monitored at weekly intervals up to 7 weeks. The growth of *M. maritima* calli displayed a normal sigmoidal curve having log, lag, and stationary phases from 0 to 1, 1 to 5, and 5 to 7 weeks, respectively. The results disclosed that the biomass of the callus intensified slowly with an increase in the cultivation period. The maximum fresh weight (FW) and dry weight (DW) of the calli were obtained after 5 and 6 weeks of cultivation, respectively (Figure 2b).

#### 2.1.2. Shoot Multiplication

Nodal explants of *M. maritima* cultivated on MS without phytohormones (control medium) did not produce axillary shoots and died after 4 weeks of incubation. Shoots developed from explants of *M. maritima* after 2 weeks of culturing on MS with different 6-BA and 6-KN (N^6^-furfuryladenine) combinations. Significant (*p* < 0.05) differences were noted in multiple shoot production among the different 6-BA and 6-KN combinations (Table 1). The medium with 2 µM 6-BA and 4 µM 6-KN had the best shoot induction rate (91.1%) with a mean of 13.4 shoots (Figure 3b, Table 1). However, the medium containing 6-BA and 6-KN at 8 µM was the least effective in promoting the induction of multiple shoots from explants of *M. maritima*.

Various 6-BA and NAA combinations were studied for axillary shoot production. Approximately 38.4–72.0% of nodal explants produced axillary shoots with an average shoot number of 1.7–6.3 after 4 weeks of cultivation on MS with different 6-BA and NAA combinations. The rate of shoot initiation and induced shoot number per node were increased in the medium augmented with 1 µM of NAA and all three levels of 6-BA. However, shoot production decreased when the *M. maritima* nodal segments were cultivated on MS augmented with 2 µM of NAA and all three levels of 6-BA. The best combination for axillary shoot production was 8 µM 6-BA and 1 µM NAA (Table 2). A combination of 6-KN and NAA was also studied for massive axillary shoots regeneration from the nodal segments of *M. maritima*. Approximately 53.7–80.1% of nodal explants produced axillary shoots with an average shoot number of 2.3–8.4 after 4 weeks of cultivation on MS with various 6-BA and NAA combinations. Out of the six combined treatments studied, the medium containing 8 µM 6-KN and 1 µM NAA induced the highest shoot number (8.4 per node) with an induction rate of 80.1% (Table 2). Calli formed from the base of the nodal segments when they were cultivated on MS augmented with combinations of 6-BA or 6-KN and NAA (Figure 3c). However, the calli induced on all media were unable to differentiate into shoots.

#### 2.1.3. Shoot Elongation and Root Induction

Shoots (4 weeks old) obtained from the multiplication medium were elongated on MS medium without any PGR (Figure 3d). The shoots developed adventitious roots after 7 days of cultivation on rooting medium 1/2 MS plus Indole-3-butyric acid (IBA 0–8 µM). The rooting frequency of *M. maritima* was 56.7% in the half-strength medium. The rooting rate, number of adventitious roots produced, and mean length varied (Table 3). The inclusion of 1–8 µM IBA in the half-strength medium stimulated adventitious rooting compared to the control (devoid of hormone). The highest number of adventitious roots number (16.2) and their length (7.6 cm) were significantly higher on rooting medium ½ MS with 2 µM IBA (Figure 3e,f, Table 3) compared with other treatments. A further increase in the level of IBA declined the number of formed adventitious roots per explant of *M. maritima* and the root growth. However, the rate of root induction was not significantly (*p* < 0.05) different (Table 3).

### 2.2. Chemical Composition

The total amount of phenolic (TAP) and flavonoid (TAF) in the *M. maritima* tissue extracts was estimated using colorimetric methods, and the data are provided in Table 4. The shoots (41.98 mg GAE/g of extract) contained the highest TAP, followed by the seedlings (19.40 mg GAE/g of extract) and calli (11.36 mg GAE/g of extract). Regarding the TAF, we observed the same order: shoots (1.76 mg RE/g of extract) > seedlings (0.73 mg RE/g of extract) > calli (0.41 mg RE/g of extract).

Both positive and negative ion electrospray ionization mass spectra were acquired over the mass range 100–1500 Da. Data acquisition was performed in the data-dependent acquisition (DDA) mode. The application of this technique allows the determination of the exact molecular mass, and the fragments were also recoded with high accuracy. The identification of the detected compounds in the calli, shoot, or seedling extracts of *M. maritima* was based on their fragmentation patterns and chromatographic behaviors (The figures are in Supplementary file: Figures S1–S6). The results showed that, in some cases, the negative mode was more sensitive for the identification of these compounds.

The number of detected compounds and chemical composition in the extracts of the shoots and seedlings of *M. maritima* were similar. In total, 39 compounds were tentatively or unambiguously detected in the shoot extract (Table 5), and 33 compounds were found in the seedling extract (Table 6). Only 18 components were detected in the callus extract (Table 7).

Further, pantothenic; undecanedioic; dodecanedioic; tetradecanedioic; hexadecanedioic; stearidonic acid and their derivatives; abscisic acid; intermedine or lycopsamine and their *N*-oxide; heliotrine; caffeic acid and its derivatives; rosmarinic acid and its di-*O*-hexoside isomers; 3-*O*-methylrosmarinic acid; apigenin-*C*-pentoside-*O*-hexoside; echimidine or heliosupine; quercetin and isoquercitine; 3,4-dihydrocinnamic acid derivatives; and di-, tri-, tetra-, pentamethoxy(iso)flavone isomers were assigned to the extracts.

**Figure 4:**
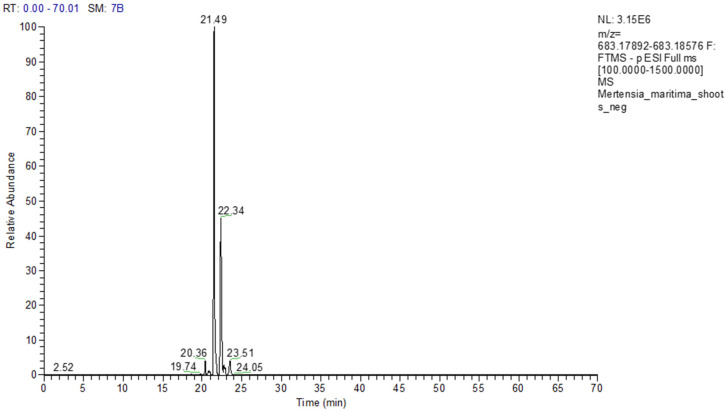
Extracted Ion Chromatogram (XIC) of rosmarinic acid di-*O*-hexoside isomers at *m*/*z* 683.1823 ([M − H]^−^) in the shoots extract.

**Figure 5:**
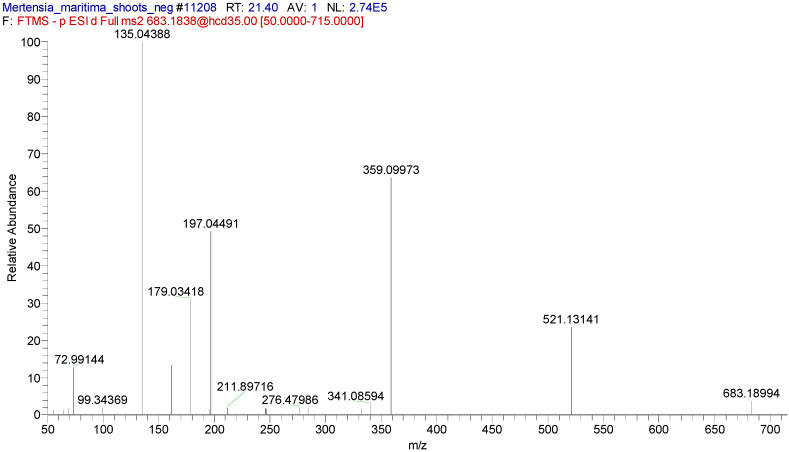
MS2 spectrum of rosmarinic acid di-*O*-hexoside isomer 1 at 21.49 min (ESI-).

**Figure 6:**
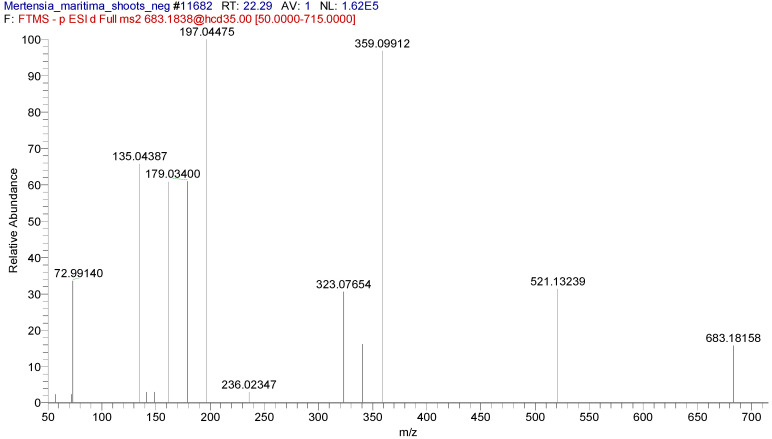
MS2 spectrum of rosmarinic acid di-*O*-hexoside isomer 2 at 22.34 min (ESI-).

In some cases, two or three isomers of compounds could be tentatively identified in the extracts. For example, two isomers of rosmarinic acid di-*O*-hexoside were identified in the shoot extract. A typical extracted ion chromatogram of these compounds at m/z 683.1823 and Figure 4, Figure 5 and Figure 6 shows the MS2 spectra (negative ion mode).

### 2.3. Biological Activities

The antioxidant activities of *M. maritima* calli, shoot, or seedling extracts were evaluated using different test systems, and the results are provided in Table 8. The best antioxidant capacity was obtained in the shoots, followed by the seedlings and calli in the radical scavenging ABTS (2,2-azino-bis(3-ethylbenzothiazoline-6-sulphonic acid) and DPPH (2,2-diphenyl-1-picrylhydrazyl), reducing power FRAP (ferric reducing antioxidant power) and CUPRAC (cupric reducing antioxidant capacity), and phosphomolybdenum (PBD) assays. However, the order was seedlings > calli > shoots with regards to the MCA (metal chelating ability).

The enzyme inhibitory effects of *M. maritima* calli, shoot, or seedling extracts were investigated against AChE (acetylcholinesterase) and BChE (butylcholinestrase), tyrosinase, and amylase. Values are presented in Table 9. The strongest AChE inhibition properties were obtained by the calli, followed by seedlings and shoots. However, the tested samples were ranked as seedlings > shoots > calli in the BChE inhibition assay. Regarding the antityrosinase inhibition ability, the strongest ability was detected in the seedlings (IC_50_: 0.74 mg/mL), followed by the calli (IC_50_: 0.80 mg/mL) and shoots (IC_50_: 0.87 mg/mL). The anti-amylase activities of the samples were almost the same (IC_50_: 1.40–147 mg/mL).

## 3. Discussion

Callus induction and axillary shoot multiplication are significant in vitro technologies for the rapid commercial production of plantlets and bioactive metabolites. Leaf explants from *M. maritima* initiated calli only on PGR (6-BA and NAA) supplemented MS medium. A combination of PGRs such as cytokinin and auxin are generally used to induce callus initiation in *M. maritima* [5] and several other Boraginaceae members such as *Alkanna orientalis* (L.) Boiss. and *A. sieheana* Rech. f. [8], *Arnebia euchroma* (Royle) Jonst. [9], *Arnebia hispidissima* (Lehm.) A. DC. [10], *Echium italicum* L. [11], *Eritrichium sericeum* (Lehm.) A. DC. [12], *Onosma bulbotrichum* [13], and *Onosma sericeum* Willd [14]. In this study, the highest callus proliferation was attained after 5 weeks of cultivation under a 16-h photoperiod on MS medium augmented with 4 µM of each 6-BA and NAA. Fedoreyev et al. [5] also obtained maximum callus growth in *M. maritima* on W_B/A_ medium containing 2.2 µM 6-BA and 10.8 µM NAA after 30 days of cultivation in the dark. However, there was a considerable difference in the optimal levels of 6-BA and NAA. The differences in the optimal concentration of 6-BA and NAA required for the best callus growth may be due to culture conditions, callus type, and growth media.

Nodal explants of *M. maritima* failed to regenerate in MS medium lacking PGR. This is consistent with an earlier study of *M. maritima* [6]. Similar results have been reported for Boraginaceae members such as *Hackelia venusta* (Piper) H.St.John [15] and *Trichodesma indicum* (Linn) R. Br. [16]. Axillary shoot multiplication can be achieved by supplementing the growth media with optimal levels of PGRs. Cytokinin is a class of PGR that promotes axillary shoot multiplication by antagonizing apical shoot dominance. Nodal segments of *M. maritima* cultivated on medium with 6-BA and 6-KN produced axillary shoots (Table 1). Cytokinins (for shoot production) and their concentrations were chosen based on our previous study [6]. The explants cultivated on medium with 2 µM 6-BA and 4 µM 6-KN developed more shoots (13.4) than those on media containing the other five 6-BA and 6-KN combinations (Table 1). The combinations of two cytokinins (6-BA and 6-KN) were found to be effective in the shoot regeneration of Boraginaceae members [10,17]. Kumar and Rao [17] reported that *Heliotropium indicum* L. axillary buds inoculated on medium with 4.7 µM 6-KN and 2.2 µM 6-BA developed 11.8 shoots.

Several studies have shown that media with cytokinin and auxin enhance shoot proliferation in numerous members of the Boraginaceae family, including *M. maritima* [6,16,17,18,19]. The ratio of cytokinin and auxin is crucial for the production of multiple shoots. In general, high cytokinin/auxin ratio promotes shoot proliferation in many plants [20]. In this study, supplementing MS with a high level of 6-BA or 6-KN (8 µM) and low level of NAA (1 µM) yielded the maximum number of shoots. However, the incorporation of a high concentration of NAA (2 µM) in media containing 6-BA or 6-KN inhibited shoot proliferation. Similar results have been described for *A. hispidissima* [18], *H. indicum* [17], and *T. indicum* [16]. The MS added with 8 µM 6-KN and 1 µM NAA produced the maximum (8.4) number of shoots (Table 2). This number was lower than that reported for oyster plant [6]. Maximal shoot production was 17.7 shoots per node for *M. maritima* and was stimulated by 4 µM TDZ and 1 µM NAA [6].

The incorporation of IBA in ½ MS enhanced the rooting response of the explants (Table 3). IBA was chosen for in vitro rooting because it has been reported to be beneficial for adventitious root induction in *M. maritima* [6] and other Boraginaceae members [10,16,17,18]. However, the rooting response of shoots under in vitro conditions varied with the IBA concentration. The optimal level of IBA required for the rooting of micro shoots depends on the rooting medium strength, the composition of shoot production medium, plant species, and genotype [20]. In this study, the best rooting was attained with 2 µM IBA in *M. maritima*. However, the rooting of *M. maritima* was the most effective in half-strength medium with 4 µM IBA [6]. MS with 9.8 µM IBA exhibited the best rooting in *A. hispidissima* [18], half-strength medium with 0.49 µM IBA showed the best rooting in *H. indicum* [17], and MS with 2.46 µM IBA displayed the best rooting in *T. indicum* [16].

Phenolics are unique compounds with significant biological properties, including antioxidant, anti-microbial, and anti-cancer activities [21]. In this context, the determination of the TAP in plant extracts is the first step; for this purpose, a colorimetric method was developed by Folin and Ciocalteu [22]. The TAP in the shoots was 216.4% and 369.5% higher than in seedlings and calli, respectively. The TAP in *M. maritima* shoots extract was also larger (41.98 mg GAE/g of extract) than in calli (9.20 GAE/g of extract), in vivo leaves (16.06 GAE/g of extract), stems (14.55 GAE/g of extract), inflorescences (21.70 GAE/g of extract) and roots (3.17 GAE/g of extract) of *Heliotropium indicum* [23], flowers (18.43 GAE/g of extract) and roots (13.11 GAE/g of extract) of *Cynoglossum creticum* [24], and aerial parts (32.7 GAE/g of extract) [25] and roots (11.45 GAE/g of extract) of *Symphytum anatolicum* [26]. The TAP in the shoots was 241.1% and 429.3% higher than in seedlings and calli, respectively. However, TAP in *M. maritima* shoot tissues was lower (1.76 mg RE/g of extract) compared to in vivo leaves (4.39 RE/g of extract) and flowers (21.77 RE/g of extract) of *C. creticum* [24], aerial parts (20.9 RE/g of extract) of *Cynoglottis barrelieri* [25], leaves (3.32 QE/g of extract), stems (3.23 QE/g of extract) and inflorescences (4.90 QE/g of extract) of *H. indicum* [23], and aerial parts (13.3 RE/g of extract) [25] and roots (2.74 RE/g of extract) of *S. anatolicum* [26]. Folin and Ciocalteu method, one of the most popular, is simple and requires inexpensive reagents. However, in recent times, there have been some concerns regarding the use of colorimetric methods, and some authors reported that these assays do not accurately reflect the levels of phytochemicals in extracts. This fact is related to the reduction of the Folin–Ciocalteu reagent by not only phenolics but also non-phenolic compounds [27]. In this sense, at least one chromatographic technique (HPLC, LC-MS, or LC-MS/MS) is important for determining accurate levels of bioactive compounds. Hence, we determined the chemical profiles of *M. maritima* extracts using the UHPLC-MS/MS technique.

The oyster plant is eaten by the Iñupiat of Alaska. It is cultivated as an edible plant in southwestern France and Northern Scotland [4] for their fragrant leaves. Thus, knowledge of its chemical composition is necessary. Pyrrolizidine alkaloids are one of the main bioactive compounds of Boraginaceae members [28]. For the first time, seven pyrrolizidine alkaloids, such as echimidine, heliosupine, heliotrine, intermedine or lycopsamine, and their N-oxides were identified in *M. maritima* extracts by UHPLC-MS/MS (Table 5, Table 6 and Table 7). The occurrence of these compounds was also disclosed in numerous members of the Boraginaceae family, including *Mertensia* species [3,28,29]. Four and six fatty acids were identified in callus and tissue extracts of *M. maritima* by UHPLC-MS/MS (Table 5–7). Among the fatty acids detected, α-linolenic acid and stearidonic acid were reported in *M. maritima* [6]. In this study, undecanedioic, dodecanedioic, tetradecanedioic, and hexadecanedioic acids were identified in *M. maritima*, for the first time. Of the phenolic compounds detected, only rosmarinic acid was reported in the callus extract of *M. maritima* [5]. Whereas other phenolic compounds were found in members of the Boraginaceae [30,31,32,33,34].

**Figure 7:**
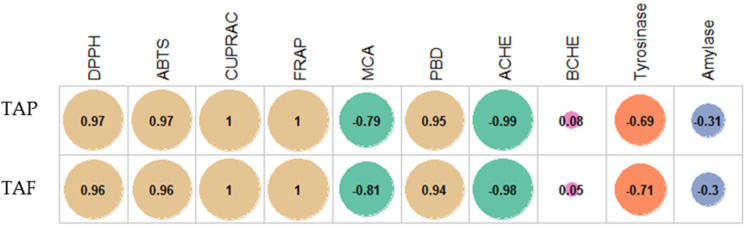
Pearson correlation between TAP, TAF and biological activities (*p* < 0.05). TAP: Total amount of phenolic; TAF: Total amount of flavonoid; DPPH: 2,2-Diphenyl-1-picrylhydrazyl; ABTS: 2,2-Azino-bis(3-ethylbenzothiazoline-6-sulphonic acid); CUPRAC: Cupric reducing antioxidant capacity; FRAP: Ferric reducing antioxidant power; MCA: Metal chelating ability; PBD: Phosphomolybdenum; AChE: Acetylcholinesterase; BChE: Butylcholinestrase.

In the last few decades, the terms “antioxidant” and “oxidative stress” have become popular terms in the scientific community [35]. This fact could be explained by the role of oxidative stress in the progression of chronic and degenerative diseases [36]. In light of this information, we determined the antioxidant profiles of *M. maritima* extracts using different chemical methods. Except for the metal chelation assay, the best antioxidant properties were obtained in the shoot, followed by the seedling and callus. The order is in line with the levels of TAP and TAF. These findings showed that phenolics were the main contributors of the antioxidant capacities of the *M. maritima* extract. This fact also was confirmed by correlation analysis, and the results are given in Figure 7. In addition, this approach was also confirmed by several authors who reported a linear relationship between total phenolics and antioxidant properties [37,38,39]. In addition, individual compounds, including caffeic acid [40], rosmarinic acid [41], and rutin [42], have been reported as significant antioxidants. The contents of these compounds are relatively high in members of the Boraginaceae family, including *M. maritima* [5,30,32,33,34]. Phenolic compounds with one or more hydroxyl groups are effective hydrogen or electron donors. Regarding the metal chelating ability, the contradictory results could be explained by the presence of non-phenolic chelators in *M. maritima* shoot extracts, including polysaccharides, peptides, or vitamin C [43].

According to WHO reports, diseases such as heart disease, stroke, chronic pulmonary disease, Alzheimer’s disease, and diabetes mellitus are the biggest killers worldwide [44]. The inhibition of cholinesterase increases the level of acetylcholine, which could enhance the memory capacity in patients with Alzheimer’s [45]. Similarly, the inhibition of carbohydrate-hydrolyzing enzymes can retard the increase in blood glucose levels in patients with diabetes [46]. Hence, several compounds are synthetically produced as enzyme inhibitors in the pharmaceutical industry. However, several studies have reported that synthetic compounds have unpleasant side effects [47,48]. Thus, enzyme inhibitors from natural sources are needed to replace these synthetic ones. In recent investigations, several researchers reported enzyme inhibition properties of several Boraginaceae members such as, *Alkanna sfikasiana* [49], *Cynoglossum creticum* [24], *Cynoglottis barrelieri* [25], *Echium confusum* [50], *Onosma aucheriana*, *Onosma sieheana*, *Onosma frutescens*, *Onosma stenoloba*, and *Onosma sericea* [51,52] *Symphytum anatolicum* [25,26]. In the present study, the enzyme inhibitory effects of *M. maritima* extracts were investigated using different enzymes. Among the three *M. martima* tissue extracts studied, calli extracts exhibited ideal AChE (IC50: 0.74 mg/mL) inhibition, seedlings extracts exhibited ideal BChE (IC50: 0.74 mg/mL) inhibition and tyrosinase (IC50: 0.74 mg/mL) inhibition, while all three extracts exhibited similar amylase (IC50: 1.40–1.47 mg/mL) inhibition activity (Table 9). The IC50 values attained from tyrosinase and amylase inhibition assays were lower compared to several other Boraginaceae members [51,52]. We observed different results for each enzyme inhibition ability. The observed enzyme inhibitory effects could be explained by the chemical profiles of the extracts. Some compounds in the chemical profiles, including caffeic acid [53,54], rosmarinic acid [55,56], and rutin [57,58] have been reported as significant enzyme inhibitor agents in previous studies. To our knowledge, the present study is the first report on the enzyme inhibitory effects of *M. maritima* extracts, and these findings could provide valuable contributions to the scientific community.

## 4. Materials and Methods

### 4.1. In Vitro Propagation

#### 4.1.1. Surface Disinfection, Media, and Culture Conditions

Leaf and node explants of *M. maritima* used in callus and shoot regeneration studies were harvested from plants cultivated in a greenhouse. The shoots were cautiously rinsed with water for 15 min and then surface decontaminated in ethanol (70% *v*/*v*) for 90 s; rinsed twice in sterilized distilled water and sodium hypochlorite solution (2% *v*/*v*) with 2–3 drops of nonionic detergent (Tween 20) for 10 min; washed three to five times in sterilized distilled water and ethanol (70% *v*/*v*) for 90 s; and rinsed twice in sterilized distilled water [6]. The cultivation media consisted of MS [59] minerals, vitamins, PGRs, sugar (30 g/L sucrose), and solidifying agent (8 g/L plant agar). The pH of all cultivation media was adjusted to 5.75 ± 0.25 before being autoclaved for 20 min at 121 °C. Leaf (callus), callus (proliferation), node (multiple shoot), micro shoot (elongation), and well-developed shoot (rooting) explant cultures were incubated for 5, 7, 4, 5, and 6 weeks, respectively, at 22–24 °C under a 16-h photoperiod (55 ± 5 µmol m^−2^ s^−1^).

#### 4.1.2. Callus Induction

Leaf explants (5–10 mm) were prepared from surface-disinfected *M. maritima* shoots placed on medium with 0, 2, or 4 µM 6-BA in combination with 0, 2, 4, or 8 µM of NAA for callus formation. To study the growth of *M. maritima* callus, approximately 50 mg of callus was inoculated on a growth medium containing 4 µM of each 6-BA and NAA. Each callus initiation or callus growth treatment consisted of 15 or 25 leaf or callus explants with three replicates. Callusing percentages and fresh and dry weights of calluses were recorded. 

#### 4.1.3. Shoot Multiplication

Node explants (approximately 5 mm) were prepared from surface-disinfected *M. maritima* shoots placed on medium with 6-BA and 6-KN (Table 1), 6-BA and NAA, or 6-KN and NAA combinations (Table 2) for multiple shoot induction. Each multiple shoot production treatment consisted of 25 nodes with three replicates. Shooting percentages and shoot numbers were recorded.

#### 4.1.4. Shoot Elongation and Root Induction

For elongation of *M. maritima* shoots, in vitro-induced shoot masses obtained from medium with 2 µM 6-BA and 4 µM 6-KN were transferred to PGRs-free growth medium. After 5 weeks, well-developed shoots (≥2 cm in height) were cultured on medium ½ MS with 0–8 µM IBA for rooting. Each root induction treatment consisted of 25 shoots with three replicates. Rooting percentages, number, and length of roots were recorded.

### 4.2. Phytochemical Analysis

#### 4.2.1. Extract Preparation

Calli (obtained from the medium with 4 µM each of 6-BA and NAA), leaves (obtained from shoots developed in PGR-free medium), and seedlings (obtained from the greenhouse) were collected after 5 weeks of cultivation; they were lyophilized and then powdered. The samples (50 mg) were extracted with methanol (80%) using an Ultraturrax at 6000 g for 20 min. After, filtration solvents were removed using a rotary evaporator and kept at plus 4 °C until further use.

#### 4.2.2. Determination of TAP and TAF

TAP in calli, shoot, or seedling extracts of *M. maritima* was assessed using the Folin–Ciocalteu method, as adapted by Slinkard and Singleton [60] and calculated as gallic acid equivalent (GAE). TAF in calli, shoot, or seedling extracts of *M. maritima* was estimated using the aluminum chloride (AlCl_3_) method, as adapted by Zengin et al. [61] and was calculated as RE. All assays were performed in three analytical replications.

#### 4.2.3. Identification and Quantification of Phytochemicals

The qualitative analysis of phytochemicals in calli, shoot, or seedling extracts of *M. maritima* was carried out using an UHPLC instrument (Dionex Ultimate 3000 RS, Thermo Scientific, MA, 01001 USA) connected to a mass spectrometer (Q Exactive Orbitrap, Thermo Scientific, USA). Thermo Accucore C18 column (100 mm length × 2.1 mm column I.D., 2.6 µm) was used for chromatographic separation [62]. Analytical details are presented in the supplementary material.

### 4.3. Biological Activities

#### 4.3.1. Antioxidant Assay

ABTS, DPPH, FRAP, CUPRAC, MCA, and PBD assays were conducted to estimate the antioxidant ability of calli, shoot, or seedling extracts of *M. maritima* and standards (Trolox and EDTA) [63]. All assays were performed in three analytical replications.

#### 4.3.2. Enzyme Inhibitory Assay

Amylase-, AChE-, BChE-, and tyrosinase-inhibitory activity of calli, shoot, or seedling extracts of *M. maritima* was determined according to Uysal et al. [63]. All assays were performed in three analytical replications.

### 4.4. Statistical Analysis

Data obtained such as callusing, shooting, rooting, TAP, TAF, and antioxidant and enzyme inhibition were analyzed in SAS version 9.1. Analysis of variance and Duncan (at 5% level) were used to test the influence and significance of treatments, respectively. For correlation analysis (Pearson coefficient), R software v. 3.5.1 was used.

## 5. Conclusions

An improved in vitro micropropagation method for *M. maritima* was established. This in vitro callus and shoot proliferation method will be useful for the extraction of bioactive metabolites from *M. maritima*. The synthesis of bioactive metabolites in *M. maritima* cell and shoot cultures is affected by chemical factors. Thus, further research on the optimization of growth media, culture environment, and elicitation are needed to maximize bioactive metabolite production in *M. maritima* in vitro cultures.

## Figures and Tables

**Table 1:** Influence of 6-BA and 6-KN combinations on shoot proliferation of *M. maritima* after 4 weeks.

6-BA (µM)	6-KN (µM)	Shoot Induction (%)	Number of Shoots
0	0	0.0 ± 0.0 i	0.0 ± 0.0 f
2	2	54.4 ± 2.1 g	4.6 ± 0.9 d
4	2	62.1 ± 2.9 e	7.2 ± 1.2 c
8	2	68.3 ± 2.5 d	6.4 ± 1.1 c
2	4	91.1 ± 2.5 a	13.4 ± 1.9 a
4	4	83.4 ± 3.2 b	9.8 ± 1.6 b
8	4	71.0 ± 2.9 c	5.9 ± 1.4 c
2	8	58.6 ± 2.4 f	6.7 ± 1.2 c
4	8	52.2 ± 2.6 g	4.1 ± 1.4 de
8	8	39.3 ± 2.3 h	2.9 ± 0.9 e

Mean ± SD with different letters (a–i) are significantly different at *p* < 0.05 (DMRT). 6-BA: N^6^-benzyladenine; 6-KN: Kinetin.

**Table 2:** Effects of PGRs combinations on shoot multiplication of *M. maritima* after 4 weeks.

6-BA (µM)	6-KN (µM)	NAA (µM)	Shoot Induction (%)	Number of Shoots
0	0	0	0.0 ± 0.0 j	0.0 ± 0.0 j
2	0	1	45.9 ± 2.6 h	3.6 ± 0.9 ef
4	0	1	64.8 ± 3.3 cd	4.7 ± 0.7 c
8	0	1	72.0 ± 4.0 b	6.3 ± 1.1 b
2	0	2	38.4 ± 3.9 i	1.7 ± 0.5 i
4	0	2	41.1 ± 3.8 i	2.7 ± 0.7 gh
8	0	2	51.9 ± 2.8 g	3.1 ± 0.8 efg
0	2	1	59.6 ± 3.6 f	2.9 ± 0.8 fgh
0	4	1	67.6 ± 2.7 c	5.7 ± 0.7 b
0	8	1	80.1 ± 3.1 a	8.4 ± 0.9 a
0	2	2	53.7 ± 3.4 g	2.3 ± 0.5 hi
0	4	2	60.3 ± 3.6 ef	3.8 ± 0.7 de
0	8	2	63.0 ± 3.8 de	4.4 ± 0.5 cd

Mean ± SD with different letters (a–j) are significantly different at *p* < 0.05 (DMRT). PGRs: Plant growth regulators; 6-BA: N^6^-benzyladenine; 6-KN: Kinetin; NAA: 1-Naphthylacetic acid.

**Table 3:** Rooting of *M. maritima* shoots after 6 weeks of incubation on half strength MS medium with IBA.

IBA (µM)	Root Induction (%)	Number of Roots	Root Length (cm)
0	56.7 ± 2.3 c	3.1 ± 0.8 d	1.6 ± 0.4 d
1	78.8 ± 3.9 b	6.8 ± 1.1 c	3.2 ± 0.5 c
2	100 ± 0.0 a	16.2 ± 1.9 a	7.6 ± 0.6 a
4	97.5 ± 3.1 a	11.3 ± 1.2 b	5.1 ± 0.6 b
8	99.1 ± 1.1 a	7.5 ± 1.3 c	4.7 ± 0.7 b

Mean ± SD with different letters (a–d) are significantly different at *p* < 0.05 (DMRT). MS: Murashige and Skoog; IBA: Indole-3-butyric acid.

**Table 4:** TAP and TAF in the *M. maritima* tissue extracts.

Samples	TAP (mg GAE/g of Extract)	TAF (mg RE/g of Extract)
Callus	11.36 ± 0.16 c	0.41 ± 0.04 c
Shoots	41.98 ± 0.37 a	1.76 ± 0.04 a
Seedling	19.40 ± 0.14 b	0.73 ± 0.04 b

TAP: Total amount of phenolic; TAF: Total amount of flavonoid; GAE: Gallic acid equivalent; RE: Rutin equivalent. Values are expressed as mean ± SD. Different letters indicate significant differences in the extracts (*p* < 0.05).

**Table 5:** Chemical inventory of shoots of *Mertensia maritima.*

No.	Name	Formula	Rt	[M + H]^+^	[M − H]^−^	Fragment 1	Fragment 2	Fragment 3	Fragment 4	Fragment 5
1	Pantothenic acid	C_9_H_17_NO_5_	6.09	220.11850		202.1075	184.0970	116.0345	90.0554	72.0451
2	Intermedine or Lycopsamine	C_15_H_25_NO_5_	7.67	300.18110		156.1020	138.0915	120.0811	94.0656	82.0657
3	Intermedine N-oxide or Lycopsamine N-oxide	C_15_H_25_NO_6_	12.37	316.17602		226.1438	172.0967	155.0942	138.0914	94.0655
4	Heliotrine or isomer	C_16_H_27_NO_5_	14.55	314.19675		156.1019	138.0915	120.0810	96.0812	
5	Caffeic acid	C_9_H_8_O_4_	15.17		179.03444	135.0439	107.0485			
6	Caffeoylshikimic acid	C_16_H_16_O_8_	18.50		335.07670	179.0340	161.0233	135.0439	111.0438	93.0330
7	Riboflavin	C_17_H_20_N_4_O_6_	19.07	377.14611		359.1364	243.0878	200.0826	172.0868	69.0343
8	Rosmarinic acid di-*O*-hexoside isomer 1	C_30_H_36_O_18_	21.49		683.18234	521.1293	359.0996	197.0449	179.0341	135.0439
9	Rosmarinic acid di-*O*-hexoside isomer 2	C_30_H_36_O_18_	21.49		683.18234	521.1324	359.0991	197.0448	179.0340	135.0439
10	Apigenin-*C*-pentoside-*O*-hexoside	C_26_H_28_O_14_	21.67	565.15574		403.1020	385.0918	367.0815	337.0709	283.0602
11	Rosmarinic acid *O*-hexoside isomer 1	C_24_H_26_O_13_	21.69		521.12952	359.0779	341.0863	179.0340	161.0232	135.0439
12	Rosmarinic acid *O*-hexoside isomer 2	C_24_H_26_O_13_	22.43		521.12952	359.0774	341.0880	179.0339	161.0232	135.0439
13	Echimidine or Heliosupine	C_20_H_31_NO_7_	22.63	398.21788		220.1330	120.0811	83.0498		
14	Echimidine or Heliosupine	C_20_H_31_NO_7_	23.02	398.21788		380.2054	220.1331	120.0810	83.0497	
15	Rosmarinic acid *O*-hexoside isomer 3	C_24_H_26_O_13_	23.42		521.12952	359.0753	341.0877	179.0340	161.0232	135.0439
16 ^1^	Isoquercitrin (Quercetin-3-*O*-glucoside)	C_21_H_20_O_12_	23.45		463.08765	301.0356	300.0276	271.0250	255.0299	151.0022
17 ^1^	Rutin (Quercetin-3-*O*-rutinoside)	C_27_H_30_O_16_	23.53		609.14557	300.0277	271.0249	255.0296	178.9977	151.0024
18	Methyl caffeate	C_10_H_10_O_4_	24.71	195.06574		163.0390	145.0285	135.0442	117.0337	107.0495
19	Rosmarinic acid (Labiatenic acid)	C_18_H_16_O_8_	24.73		359.07670	197.0448	179.0340	161.0232	135.0438	72.9915
20	Kaempferol-3-*O*-rutinoside (Nicotiflorin)	C_27_H_30_O_15_	25.38		593.15065	285.0407	284.0329	255.0298	227.0345	
21	Abscisic acid	C_15_H_20_O4	25.81		263.12834	219.1383	204.1149	201.1278	152.0830	151.0751
22	Ethyl caffeate	C_11_H_12_O_4_	26.52	209.08139		163.0390	145.0285	135.0443	117.0338	89.0391
23	3-*O*-Methylrosmarinic acid	C_19_H_18_O_8_	26.65		373.09235	197.0448	179.0340	175.0389	160.0154	135.0439
24 ^1^	Quercetin (3,3′,4′,5,7-Pentahydroxyflavone)	C_15_H_10_O_7_	27.56		301.03483	178.9979	151.0024	121.0279	107.0123	
25	3,4-Dihydroxycinnamoyl-2-(3,4-dihydroxyphenyl) ethenol (cis isomer)	C_17_H_14_O_6_	29.08	315.08687		205.0500	163.0390	145.0285	135.0442	123.0443
26	3,4-Dihydroxycinnamoyl-2-(3,4-dihydroxyphenyl) ethenol (trans isomer)	C_17_H_14_O_6_	30.06	315.08687		205.0499	163.0390	145.0285	135.0443	123.0443
27	Undecanedioic acid	C_11_H_20_O_4_	31.36		215.12834	197.1177	153.1273	125.0959	57.0332	
28	Pentamethoxy(iso)flavone	C_20_H_20_O_7_	32.92	373.12873		358.1035	357.1026	343.0814	327.0867	312.0992
29	Dodecanedioic acid	C_12_H_22_O_4_	33.80		229.14399	211.1332	167.1430			
30	Dimethoxy(iso)flavone	C_17_H_14_O_4_	34.14	283.09704		268.0730	267.0648	239.0702	225.0550	
31	Trimethoxy(iso)flavone isomer 1	C_18_H_16_O_5_	34.81	313.10760		298.0835	297.0754	269.0809	268.0728	255.0655
32	Trimethoxy(iso)flavone isomer 2	C_18_H_16_O_5_	34.99	313.10760		298.0837	297.0760	269.0807	267.0648	252.0781
33	Tetramethoxy(iso)flavone	C_19_H_18_O_6_	35.44	343.11817		328.0934	327.0858	314.0780	313.0704	299.0905
34	Tetradecanedioic acid	C_14_H_26_O_4_	37.73		257.17529	239.1649	195.1747			
35	Dimethoxy-hydroxy(iso)flavone	C_17_H_14_O_5_	38.94	299.09195		284.0678	283.0600	256.0729	255.0648	
36	Hexadecanedioic acid	C_16_H_30_O_4_	40.78		285.20659	267.1967	223.2062			
37	Stearidonic acid methyl ester	C_19_H_30_O_2_	42.16	291.23241		259.2058	241.1961	199.1487	135.1170	93.0704
38	Stearidonic acid ethyl ester	C_20_H_32_O_2_	43.06	305.24806		259.2055	241.1955	199.1483	135.1170	93.0703
39 ^1^	α-Linolenic acid	C_18_H_30_O_2_	45.13		277.21676	259.2064	233.2268	205.1957	59.0122	

^1^ Confirmed by standard. Rt: Retention time.

**Table 6:** Chemical inventory of seedling of *Mertensia maritima*.

No.	Name	Formula	Rt	[M + H]^+^	[M − H]^−^	Fragment 1	Fragment 2	Fragment 3	Fragment 4	Fragment 5
1	Pantothenic acid	C_9_H_17_NO_5_	6.09	220.11850		202.1075	184.0970	116.0347	90.0555	72.0450
2	Intermedine or Lycopsamine	C_15_H_25_NO_5_	7.87	300.18110		156.1020	138.0915	120.0810	94.0657	82.0657
3	Intermedine N-oxide or Lycopsamine N-oxide	C_15_H_25_NO_6_	12.44	316.17602		226.1441	172.0968	155.0944	138.0915	94.0656
4	Heliotrine or isomer	C_16_H_27_NO_5_	14.55	314.19675		156.1021	138.0916	120.0806	96.0812	
5	Caffeic acid	C_9_H_8_O_4_	15.18		179.03444	135.0438	107.0489			
6	Caffeoylshikimic acid	C_16_H_16_O_8_	18.52		335.07670	179.0340	161.0232	135.0439	111.0436	93.0330
7	Riboflavin	C_17_H_20_N_4_O_6_	19.07	377.14611		359.1354	243.0877	200.0820	172.0870	69.0342
8	Rosmarinic acid *O*-hexoside isomer 2	C_24_H_26_O_13_	22.44		521.12952	359.0774	341.0889	179.0338	161.0231	135.0439
9	Echimidine or Heliosupine	C_20_H_31_NO_7_	22.63	398.21788		220.1331	120.0809	83.0498		
10	Echimidine or Heliosupine	C_20_H_31_NO_7_	22.99	398.21788		380.2090	220.1333	120.0811	83.0498	
11	Rosmarinic acid *O*-hexoside isomer 3	C_24_H_26_O_13_	23.46		521.12952	359.0769	341.0883	179.0340	161.0232	135.0439
12 ^1^	Isoquercitrin (Quercetin-3-*O*-glucoside)	C_21_H_20_O_12_	23.47		463.08765	301.0358	300.0277	271.0252	255.0296	151.0024
13 ^1^	Rutin (Quercetin-3-*O*-rutinoside)	C_27_H_30_O_16_	23.55		609.14557	300.0276	271.0249	255.0297	178.9972	151.0025
14	Methyl caffeate	C_10_H_10_O_4_	24.76	195.06574		163.0390	145.0285	135.0442	117.0338	107.0496
15	Rosmarinic acid (Labiatenic acid)	C_18_H_16_O_8_	24.80		359.07670	197.0448	179.0339	161.0231	135.0438	72.9915
16	Kaempferol-3-*O*-rutinoside (Nicotiflorin)	C_27_H_30_O_15_	25.40		593.15065	285.0405	284.0328	255.0296	227.0346	
17	Abscisic acid	C_15_H_20_O_4_	25.83		263.12834	219.1382	204.1143	201.1274	152.0833	151.0750
18	Ethyl caffeate	C_11_H_12_O_4_	26.45	209.08139		163.0390	145.0285	135.0442	117.0337	89.0390
19	3-*O*-Methylrosmarinic acid	C_19_H_18_O_8_	26.65		373.09235	197.0448	179.0339	175.0389	160.0153	135.0439
20 ^1^	Quercetin (3,3′,4′,5,7-Pentahydroxyflavone)	C_15_H_10_O_7_	27.58		301.03483	178.9981	151.0025	121.0280	107.0123	
21	3,4-Dihydroxycinnamoyl-2-(3,4-dihydroxyphenyl) ethenol (cis isomer)	C_17_H_14_O_6_	29.07	315.08687		205.0495	163.0391	145.0287	135.0444	123.0442
22	3,4-Dihydroxycinnamoyl-2-(3,4-dihydroxyphenyl) ethenol (trans isomer)	C_17_H_14_O_6_	30.07	315.08687		205.0498	163.0391	145.0286	135.0443	123.0443
23	Undecanedioic acid	C_11_H_20_O_4_	31.37		215.12834	197.1176	153.1272	125.0956	57.0331	
24	Pentamethoxy(iso)flavone	C_20_H_20_O_7_	32.92	373.12873		358.1046	357.1057	343.0808	327.0869	312.0989
25	Dodecanedioic acid	C_12_H_22_O_4_	33.80		229.14399	211.1333	167.1430			
26	Dimethoxy(iso)flavone	C_17_H_14_O_4_	34.14	283.09704		268.0731	267.0650	239.0706	225.0552	
27	Trimethoxy(iso)flavone isomer 1	C_18_H_16_O_5_	34.81	313.10760		298.0840	297.0751	269.0808	268.0738	255.0655
28	Trimethoxy(iso)flavone isomer 2	C_18_H_16_O_5_	35.00	313.10760		298.0837	297.0762	269.0817	267.0648	252.0775
29	Tetramethoxy(iso)flavone	C_19_H_18_O_6_	35.44	343.11817		328.0948	327.0864	314.0786	313.0704	299.0929
30	Tetradecanedioic acid	C_14_H_26_O_4_	37.73		257.17529	239.1647	195.1747			
31	Hexadecanedioic acid	C_16_H_30_O_4_	40.77		285.20659	267.1966	223.2061			
32	Stearidonic acid methyl ester	C_19_H_30_O_2_	42.15	291.23241		259.2060	241.1949	199.1483	135.1170	93.0703
33 ^1^	α-Linolenic acid	C_18_H_30_O_2_	45.13		277.21676	259.2064	233.2272	205.1977	59.0123	

^1^ Confirmed by standard. Rt: Retention time.

**Table 7:** Chemical inventory of callus of *Mertensia maritima.*

No.	Name	Formula	Rt	[M + H]^+^	[M − H]^−^	Fragment 1	Fragment 2	Fragment 3	Fragment 4	Fragment 5
1	Intermedine N-oxide or Lycopsamine N-oxide	C_15_H_25_NO_6_	12.47	316.17602		226.1437	172.0968	155.0940	138.0915	94.0657
2	Vanillic acid	C_8_H_8_O_4_	14.42		167.03444	152.0103	123.0437	108.0202		
3	Caffeic acid	C_9_H_8_O_4_	15.19		179.03444	135.0438	107.0489			
4	Riboflavin	C_17_H_20_N_4_O_6_	19.06	377.14611		359.1311	243.0876	200.0819	172.0867	69.0341
5	Ferulic acid	C_10_H_10_O_4_	19.91		193.05009	178.0261	149.0596	137.0232	134.0361	121.0279
6	Isoferulic acid	C_10_H_10_O_4_	20.93		193.05009	178.0263	149.0596	137.0228	134.0360	
7 ^1^	Rutin (Quercetin-3-*O*-rutinoside)	C_27_H_30_O_16_	23.55		609.14557	300.0279	271.0255	255.0288	178.9974	151.0022
8	Unidentified alkaloid	C_16_H_15_NO_5_	24.39	302.10285		284.0915	141.0699	134.0449	116.0345	88.0398
9	Rosmarinic acid (Labiatenic acid)	C_18_H_16_O_8_	24.78		359.07670	197.0449	179.0341	161.0232	135.0439	72.9915
10	Methoxy-methylcoumarin isomer 1	C_11_H_10_O_3_	25.75	191.07082		176.0468	148.0519	135.0805	131.0491	105.0702
11	Camphanic acid or isomer	C_10_H_14_O_4_	27.41		197.08139	153.0909	85.0279			
12	Methoxy-methylcoumarin isomer 2	C_11_H_10_O_3_	28.09	191.07082		176.0468	148.0519	135.0806	131.0492	105.0703
13	Undecanedioic acid	C_11_H_20_O_4_	31.35		215.12834	197.1177	153.1273	125.0959	57.0332	
14	Dodecanedioic acid	C_12_H_22_O_4_	33.81		229.14399	211.1332	167.1429			
15	Dimethoxy(iso)flavone	C_17_H_14_O_4_	34,14	283.09704		268.0729	267.0650	239.0701	225.0539	
16	Tetramethoxy(iso)flavone	C_19_H_18_O_6_	35.43	343.11817		328.0938	327.0863	314.0780	313.0704	299.0905
17	Tetradecanedioic acid	C_14_H_26_O_4_	37.73		257.17529	239.1650	195.1748			
18	Hexadecanedioic acid	C_16_H_30_O_4_	40.78		285.20659	267.1966	223.2061			

^1^ Confirmed by standard. Rt: Retention time.

**Table 8:** Antioxidant properties of the extracts (IC50 (mg/mL)).

Samples	DPPH	ABTS	CUPRAC	FRAP	PBD	Chelating
Callus	>3	>3	2.91 ± 0.01 d	1.77 ± 0.01 d	>3	1.94 ± 0.15 c
Shoots	0.57 ± 0.01 b	0.78 ± 0.01 b	0.55 ± 0.01 b	0.35 ± 0.01 b	1.60 ± 0.05 b	>3
Seedling	1.18 ± 0.01 c	1.63 ± 0.03 c	1.33 ± 0.01 c	0.81 ± 0.01 c	2.91 ± 0.07 c	1.23 ± 0.20 b
Trolox	0.06 ± 0.01 a	0.09 ± 0.01 a	0.11 ± 0.01 a	0.04 ± 0.01 a	0.52 ± 0.02 a	nt
EDTA	nt	nt	nt	nt	nt	0.02 ± 0.001 a

DPPH: 2,2-Diphenyl-1-picrylhydrazyl; ABTS: 2,2-Azino-bis(3-ethylbenzothiazoline-6-sulphonic acid); CUPRAC: Cupric reducing antioxidant capacity; FRAP: Ferric reducing antioxidant power; PBD: Phosphomolybdenum; nt: no tested. Values are expressed as mean ± SD. Different letters indicate significant differences in the extracts (*p* < 0.05).

**Table 9:** Enzyme inhibitory properties of the extracts (IC50 (mg/mL)).

Samples	AChE	BChE	Tyrosinase	Amylase
Callus	0.78 ± 0.01 b	1.92 ± 0.17 d	0.80 ± 0.01 c	1.40 ± 0.08 b
Shoots	1.21 ± 0.07 d	1.66 ± 0.08 bc	0.87 ± 0.01 d	1.45 ± 0.02 b
Seedling	0.89 ± 0.02 c	1.35 ± 0.16 b	0.74 ± 0.01 b	1.47 ± 0.05 b
Galantamine	0.003 ± 0.001 a	0.007 ± 0.002 a	nt	nt
Kojic acid	nt	nt	0.08 ± 0.001 a	nt
Acarbose	nt	nt	nt	0.68 ± 0.01 a

AChE: Acetylcholinesterase; BChE: Butylcholinestrase; nt: no tested. Values are expressed as mean ± SD. Different letters indicate significant differences in the extracts (*p* < 0.05).

## Supplementary Materials

The following are available online at https://www.mdpi.com/2223-7747/9/11/1551/s1, UHPLC methods and Figures S1–S6.
