# Supplementary material for: Screening of Bioactive Metabolites and Biological Activities of Calli, Shoots, and Seedlings of Mertensia maritima (L.) Gray
Source: Plants (Basel). 2020 Nov 12;9(11):1551. doi: 10.3390/plants9111551 (PMC7697918; doi:10.3390/plants9111551)
Supplement: Supplementary file 1 [file plants-09-01551-s001.docx]

**Screening of bioactive metabolites and biological activities in callus, shoot, and seedlings of *Mertensia maritima* (L.) Gray**

Chemical composition

The chemical compositions of the *M. maritima* methanolic extracts were determined using a Dionex Ultimate 3000RS UHPLC system. Callus, shoot, and seedling extracts were filtered through a 0.22-μm PTFE syringe filter (Labex Ltd, Hungary) before UHPLC analysis. The compounds present in *M. maritima* were separated on a Thermo Accucore C18 (100 mm × 2.1, mm i. d., 2.6 μm) column thermostated at 25 °C (± 1 °C). The eluents were water (A) and methanol (B); both were acidified with 0.1% formic acid. The flow rate was maintained at 200 µL/min. The elution gradient was isocratic: 5% B (0–3 min), a linear gradient increasing from 5% B to 100% (3–43 min); 100% B (43–61 min), a linear gradient decreasing from 100% B to 5% (61–62 min), and 5% B (62–70 min). The column was coupled to a Thermo Q Exactive Orbitrap mass spectrometer (Thermo Scientific, USA) equipped with an electrospray ionization source. MS spectra were recorded in positive and negative ion mode, respectively.

Trace Finder 3.1 software (Thermo Scientific, USA) was used for target screening. The compounds listed in the tables were identified based on our previously published works or data found in literature using the exact molecular mass, isotopic pattern, and characteristic fragment ions. In each case, the exact molecular mass, isotopic pattern, characteristic fragment ions, and retention time (min) were used to identify the compounds, which were confirmed using standards.

**Figure S1.** Total ion chromatogram of callus extract of *Mertensia maritima* in negative mode

**Figure S2.** Total ion chromatogram of callus extract of *Mertensia maritima* in positive mode

**Figure S3.** Total ion chromatogram of shoot extract of *Mertensia maritima* in negative mode

**Figure S4.** Total ion chromatogram of shoot extract of *Mertensia maritima* in positive mode

**Figure S5.** Total ion chromatogram of seedling extract of *Mertensia maritima* in positive mode

**Figure S6.** Total ion chromatogram of seedling extract of *Mertensia maritima* in negative mode
